# [Corrigendum] Enhanced antitumor activity by the combination of dasatinib and combretastatin A‑4 *in vitro* and *in vivo*

**DOI:** 10.3892/or.2024.8783

**Published:** 2024-07-24

**Authors:** Chong Zhang, Shuang-Shuang Zhou, Xiang-Rong Li, Bao-Ming Wang, Neng-Ming Lin, Lin-Yi Feng, Da-Yong Zhang, Li-Huang Zhang, Jun-Bo Wang, Jian-Ping Pan

Oncol Rep 29: 2275–2282, 2013; DOI: 10.3892/or.2013.2405

Following the publication of this article, an interested reader drew to the authors' attention that the flow cytometric (FCM) plots in Fig. 2A on p. 2278 showing the ‘Dasatinib’ and ‘CA-4’ experiments were duplicates of each other. After having re-examined their original data, and due to the overall similarity of the data, the authors have realized that these data were inadvertently assembled incorrectly in the figure. They realize that they also made a further mistake regarding the writing of the ratios of mitochondrial membrane-depolarized HO-8910 cells for these FCM plots (essentially, these were written the wrong way around): The percentage of mitochondrial membrane-depolarized HO-8910 cells should have been written as 22.50% for the dasatinib-treated cells (the centre-left FCM plot) and 15.71% for the CA-4-treated cells (centre-right plot).

A revised version of Fig. 2 now showing alternative data for the FCM experiments shown in Fig. 2A, is shown on the next page. Note that the errors made in terms of assembling the data in Fig. 2A did not greatly affect either the results or the conclusions reported in this paper, and all the authors agree with the publication of this corrigendum. The authors regret that these errors went unnoticed prior to the publication of their article, and are grateful to the Editor of *Oncology Reports* for granting them this opportunity to publish a corrigendum. Furthermore, they apologize to the readership for any inconvenience caused.

## Figures and Tables

**Figure 2.**
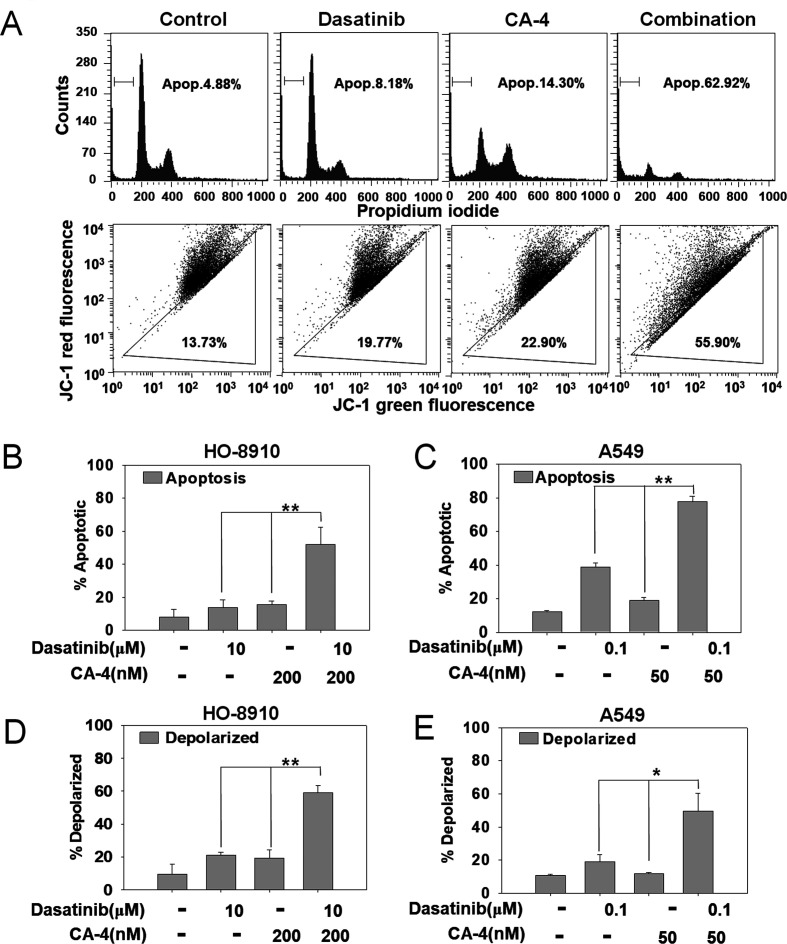
Dasatinib plus combretastatin A-4 (CA-4) causes enhanced apoptosis. (A) HO-8910 cells were treated with dasatinib (10 μM), CA-4 (200 nM) or the combination for 48 h. The cells were analyzed for apoptosis by flow cytometry and Sub-G1 contents were designed as apoptotic cells. (B) HO-8910 and (C) A549 cells in 6-well plates were exposed to compounds for 48 h and the cells were then analyzed by flow cytometry following PI staining. (D) HO-8910 and (E) A549 cells in 6-well plates were exposed to compounds for 48 h and the cells were then analyzed by flow cytometry following JC-1 staining. The experiments were repeated three times, and error bars represent standard deviation. *P<0.05, **P<0.01.

